# Falciparum malaria molecular drug resistance in the Democratic Republic of Congo: a systematic review

**DOI:** 10.1186/s12936-015-0892-z

**Published:** 2015-09-17

**Authors:** Dieudonné Makaba Mvumbi, Jean-Marie Kayembe, Hippolyte Situakibanza, Thierry L. Bobanga, Célestin N. Nsibu, Georges L. Mvumbi, Pierrette Melin, Patrick De Mol, Marie-Pierre Hayette

**Affiliations:** Biochemistry and Molecular Biology Unit, Department of Basic Sciences, School of Medicine, University of Kinshasa, Kinshasa, DR Congo; Department of Internal Medicine, School of Medicine, University of Kinshasa, Kinshasa, DR Congo; Department of Tropical Medicine and Infectious Disease, School of Medicine, University of Kinshasa, Kinshasa, DR Congo; Department of Paediatric, School of Medicine, University of Kinshasa, Kinshasa, DR Congo; Department of Clinical Microbiology, University Hospital of Liège, Liège, Belgium

**Keywords:** Malaria, DR Congo, Molecular, Drug resistance

## Abstract

**Background:**

Malaria cases were estimated to 207 million in 2013. One of the problems of malaria control is the emergence and spread of *Plasmodium falciparum* strains that become resistant to almost all drugs available. Monitoring drug resistance is essential for early detection and subsequent prevention of the spread of drug resistance by timely changes of treatment policy. This review was performed to gather all data available on *P. falciparum* molecular resistance in DR Congo, as baseline for future assessments.

**Methods:**

The search for this review was undertaken using the electronic databases PubMed and Google Scholar using the terms “malaria”, “Congo”, “resistance”, “molecular”, “antimalarial”, “efficacy”. Articles were classified based on year of collecting, year of publication, sample size and characteristics, molecular markers analysed and polymorphisms detected.

**Results:**

Thirteen articles were included and five genes have been analysed in these studies: *pfcrt*, *pfdhps*, *pfdhfr*, *pfmdr1* and K13-propeller. The majority of studies included were not representative of the whole country.

**Conclusion:**

This systematic review demonstrates the lack of molecular resistance studies in DRC. Only 13 studies were identified in almost 15 years. The MOH must implement a national surveillance system for monitoring malaria drug resistance and this surveillance should be conducted frequently and country-representative.

## Background

Malaria cases were estimated to 207 million in 2013, among with 584,000 deaths, 90 % found in Africa, Democratic Republic of Congo (DRC) and Nigeria together accounting for 40 % of the estimated global total [1]. The absence to date of an effective vaccine leaves us only chemotherapy to fight against *Plasmodium falciparum* infection, the most virulent *Plasmodium* species that infects humans. One of the problems of malaria control is the emergence and spread of *P. falciparum* strains that become resistant to almost all drugs available.

Chloroquine (CQ) was one of the most used molecules in the fight against malaria because of its cost-effectiveness, but just some years after being placed on the market, the first cases of chloroquine resistance emerged in Southeast Asia, then in Latin America before spreading to all endemic areas [2]. Later, this phenomenon was repeated for the other available drugs (proguanil, sulfadoxine–pyrimethamine, halofantrine, mefloquine). To prevent, or at least delay the onset of new resistant strains, WHO recommended in 2001 the use of drugs association and that one of the drugs be an artemisinin derivative [3]. At that time, no artemisinin resistance had been identified yet and many hopes were placed onto artemisinins. Unfortunately, in 2008 Noedl et al. reported evidence of artemisinin resistance in Western Cambodia [4]. To date, artemisinin resistance has spread to Thailand, Myanmar, Laos and Vietnam [5–7]. In 2014, Ariey et al. identified a molecular marker for artemisinin resistance [8].

Monitoring drug resistance is essential for early detection and subsequent prevention of the spread of drug resistance by timely changes of treatment policy. There are three possibilities to study drug resistance: in vitro tests, in vivo tests and study of molecular markers [9]. In vivo tests are the gold standard, as involving the three factors (human host, parasite and anti-malarial molecule), but they are difficult to implement and very expensive due to the heavy organizational machinery that must be in place (drugs, monitoring of patients for 24 or 48 days, repeated biological tests, an experienced staff).

In vitro tests allow studying the inhibition of parasite growth in culture subjected to different concentrations of an anti-malarial. Their results do not reflect a real situation where parasites can survive in vivo in presence of an optimal serum concentration of the drug, as they do not consider host immunity or drug pharmacokinetics. These tests are also financially heavy and require live parasites, qualified persons for its implementation and laboratory facilities for in vitro culture. Finally, the study of molecular markers is a good alternative although the factors related to the host and the drugs are not taken into account. They can explore a wide range of markers (previously identified as related to resistance) onto large samples and in punctual manner. So the analysis of molecular markers gives a snapshot of the situation at a given time [10]. In DRC, which is the second largest African country, CQ was widely used for years till its withdrawal in 2002, first replaced by sulfadoxine–pyrimethamine (SP), then by artemisinin-based combination therapy (ACT) [11]. Due to its size, studying malaria resistance in DRC by in vitro or in vivo methods would be very expensive and time-consuming; analysing molecular markers would be a better alternative.

This systematic review was performed to gather all data available on *P. falciparum* molecular resistance in DRC, as baseline for future assessments.

## Methods

**Fig. 1:**
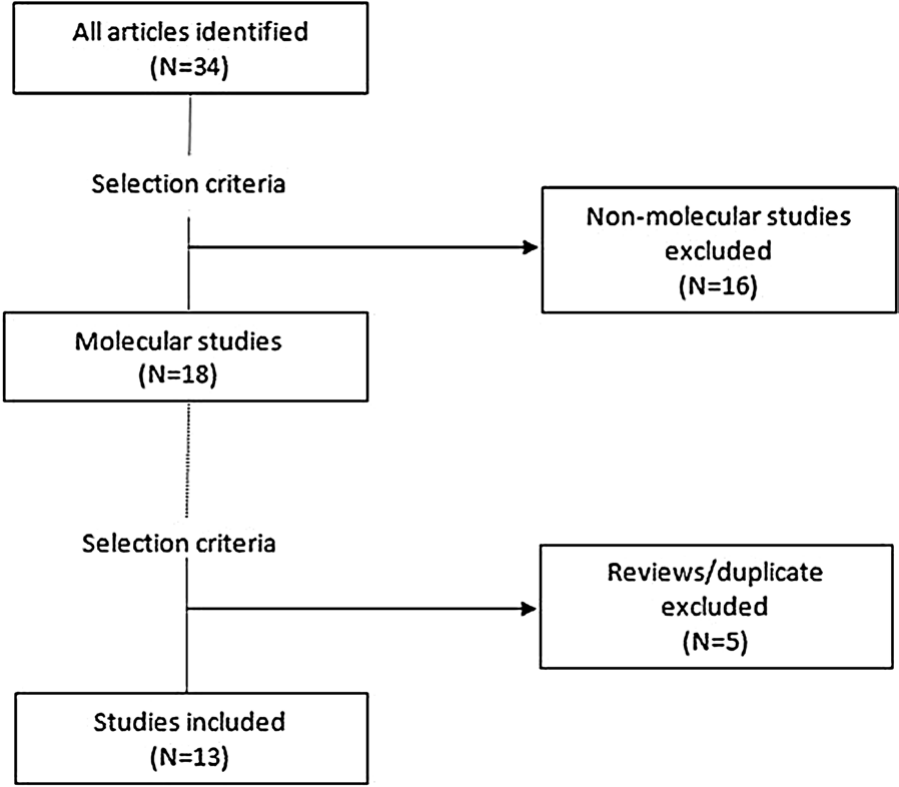
Search strategy. Twenty-one studies have been excluded after application of selection criteria

The search for this review was conducted during November 2014 and was undertaken using the electronic databases Pub Med and Google scholar. The following search terms “malaria”, “Congo”, “resistance”, “molecular”, “antimalarial”, “efficacy” were used. Additional results were obtained from the references in the articles identified through the search. Selection criteria were: (a) samples related to malaria infection in DRC; (b) study comprising analysis of one or more malaria resistance molecular marker; (c) original articles, short reports but no review articles. Full-text articles were read to check for selection criteria. An overall of 34 articles has been identified among which 16 were non-molecular studies and 5 were review articles or duplicates (based on same samples). Thirteen articles were finally included. Articles were classified based on year of collecting, year of publication, sample size and characteristics, molecular markers analyzed and polymorphisms detected. Figure 1 shows the search strategy used.

## Results

The overall of the articles included in this review were of samples collected from 1999 to 2014 [12–24]. Some of them concerned non-Congolese people but supposed to have been infected in DRC. Five genes have been analysed in these studies: the *Plasmodium falciparum* chloroquine resistance transporter gene (*pfcrt*), the dihydropteroate synthase gene (*pfdhps*), the dihydrofolate reductase gene (*pfdhfr*), the *Plasmodium falciparum* multidrug resistance 1 gene (*pfmdr1*) and the K13-propeller gene (*k13*).

The most analysed gene was *pfcrt*, linked to CQ and amodiaquine (AMQ) resistance [25, 26], and it has been studied in seven articles (53.8 %). *Pfdhps*, known to be related to sulfadoxine resistance [27] and *pfdhfr* for which evidence of point mutations conferring resistance to pyrimethamine has been made [28], have been respectively analysed in five (38.4 %) and four articles (30.7 %). Analysis concerning K13-propeller and the *pfmdr1* genes have been found, respectively in two (15.3 %) and one (7.6 %) studies. *Pfmdr1* is correlated to resistance to many drugs as mefloquine (MQ), lumefantrine (LMF), AMQ and may be to artemisinin [29–32] and *k13* is correlated to artemisinin resistance [8]. Table 1 provides a summary of the studies included in the review.

**Table 1:** Summary of the studies included in the review

	Authors	Year of collecting	Year of publication	Sample size^a^	Age	Molecular markers
1.	Andriantsoanirina et al.	1999, 2002, 2005	2010	3	–	pfcrt
2.	Severini et al.	2000	2005	32	–	pfcrt
3.	Wilson et al.	2002	2005	56	6–58 months	pfcrt
4.	Alker et al.	2002	2008	249	6–59 months	pfdhfr, pfdhps
5.	Cohuet et al.	2003–2004	2006	458	–	pfdhfr, pfdhps
6.	Swarthout et al.	2004	2006	168	6–59 months	pfdhfr, pfdhps
7.	Taylor et al.	2007	2013	179	>15 years	dhps
8.	Antonia et al.	2007	2014	180	>15 years	pfcrt
9.	Taylor et al.	2007	2014	151	>15 years	K13
10.	Mobula et al.	2008	2009	142	1–10 years	pfcrt, pfmdr1, pfdhfr, pfdhps
11.	Juliao et al.	2010	2013	12	19–55 years	pfcrt
12.	Mvumbi et al.	2010	2013	145	6–59 months	pfcrt
13.	Kamau et al.	2013–2014	2014	82	>6 years	K13

^a^Considering only samples from DRC, not the total sample size of the study

### *pfcrt* gene

On the seven studies involving the K76T mutation, four have also explored the haplotypes defined by specific mutations at amino acid positions 72–76 (related to AMQ resistance). The main findings of these articles are given in Table 2.

**Table 2:** Main findings of studies that analysed the pfcrt gene

	Authors	Year of collecting	Haplotypes found	Mutant 76T	
1.	Andriantsoanirina et al.	1999, 2002, 2005	_	3/3	100 %
2.	Severini et al.	2000	CVIET, CVMNT, SVIET	27/27	100 %
3.	Wilson et al.	2002	_	52/56	93 %
4.	Antonia et al.	2007	CVMNK, CVIET, CVMNT, CVMDK	92/166	55.4 %
5.	Mobula et al.	2008	_	88/105	83.8 %
6.	Juliao et al.	2010	CVMNK, CVIET	1/12	8 %
7.	Mvumbi et al.	2010	CVMNK, CVIET	148/198	73.2 %

### *pfdhfr* and *pfdhps* genes

Both genes are generally analysed together as they are related to SP combination susceptibility. Only one study analysed the *dhps* gene alone. Most point mutations analysed were on positions 51, 59 and 108 on *dhfr* and on positions 437 and 540 on *dhps*. Prevalence of point mutations found in these studies is presented in Table 3.

**Table 3:** Prevalence of point mutations found in dhfr/dhps genes from included studies

	Authors	Year of collecting	Dhps mutations			Dhfr mutations				Quint. mutant (dhfr and dhps)	Wild		
			A437G	K540E	Double mutants	N51I	C59R	S108N	Triple mutants	–	dhps	dhfr	both
1.	Alker et al.	2002	72 %	67 %	–	>95 %	66 %	>95 %	–	43 %	–	–	1 %
2.	Cohuet et al.	2003–2004	–	–	<20 %	–	–	–	>50 %	0.9–12.8 %	29.4–75.6 %	1–3.9 %	–
3.	Swarthout et al.	2004	0.6 %	–	45.2 %	–	–	3.2 %	46.2 %	27.1 %	30 %	7.6 %	3 %
4	Taylor et al.	2007	–	–	14.6 %	–	–	–	–	–	35.1 %	–	–
5.	Mobula et al.	2008	93.1 %	9.5 %	–	97.9 %	80.7 %	99.1 %	–	–	–	–	–

### *K13*-*propeller* gene

Recently linked to artemisinin resistance, this gene has been analysed twice in sample from DRC. The older samples are about 2007 and the recent one is of 2013–2014. None of mutations related to artemisinin resistance discovered in South-East Asia were found in DRC to date. All mutations found are shown in Table 4.

**Table 4:** Mutations found on k13 in DRC samples

	Authors	Year of collecting	Mutations found
1.	Kamau et al.	2013–2014	T493T, A578S
2.	Taylor et al.	2007	R471R, G496G, R513R, V520A, A557S, V581V, A617T, V637A

### *pfmdr1* gene

Only one study analysed this gene in Kinshasa. Five point mutations were explored on codons positions 86, 184, 1034, 1042 and 1046. The N86Y mutation was present in 66.7 % of samples studied [20].

## Discussion

During almost 15 years, only 13 molecular studies were conducted based on samples from the DRC. These studies were of samples from 3 to 458 individuals (mean = 142) and that usually were focalized to one or two geographical sites. However, three studies [18, 19, 21] were based on samples collected in the whole country as part of the Demographic and Health Survey (DHS), funded mostly by the international community [33]. Considering the size of the DRC (second largest African country with 2,345,000 km^2^) and the different patterns of malaria from one corner to another, results of most of these studies are not generalizable to the nationwide, but must be considered in their local context.

Sometimes for the same molecular markers in the same study, differences are significant in two different sites. The study of Cohuet et al. [16] illustrates well this fact: the prevalence of *dhps* double mutants (resistance to sulfadoxine) was estimated at less than 1 % in a site (Basankusu) whereas it was close to 20 % in another (Kisangani). This would like to suggest that the molecule remains active in one place while it should be replaced by another. As WHO recommends withdrawal of a drug if the prevalence of resistant is above 10 %, this suggests in that case that the drug should be replaced in one place while it should be maintained in another. This underlines the fact that making national decision about treatment policy could not be based only on “localized” studies. The Ministry of Health should initiate, regularly, anti-malarial drugs efficacy monitoring studies but with a representative sampling. This scarcity of representative studies across the country is a real handicap. Treatment policies are applied in light of the available study (and thus the geographical location concerned). Thus, when changing the SP to ACT in the DRC, one of the studies available to support the selection of the combination AS + AQ compared with AS + SP were localized to one site, Shabunda (East of the DRC) [17]. Maybe if another study was conducted simultaneously in the West of the country, the data would have been different. The main hurdle is generally the high cost of surveillance studies, in particular for molecular studies that require equipment and reagents often unavailable locally. Thus, none of molecular analysis performed for these 13 studies have been performed in a Congolese laboratory. All of the molecular analysis was carried out in Europe or in USA. Another implication of this high cost is that making these studies in a repetitive manner is very difficult. Yet the WHO experts recommend that the surveillance of the resistance must be carried out every 2 years in endemic countries [34].

The various data existing on the *pfcrt* gene confirm the decline of CQ resistant strains after its withdrawal. The latest available figures (which only concern Kinshasa) revealed a prevalence of 73.2 % [23], whereas previous studies before chloroquine replacement in 2002 were giving values beyond 90 % [12–14]. Unfortunately, as mentioned above, it is difficult to correctly compare these studies because of the differences in the applied methodology (essentially the sampling and the study location). It is important to notice that none of the studies concerning the *pfcrt* gene have detected the presence of the SVMNT haplotype (linked to AMQ resistance) in the DRC. As this haplotype was recently found in Angola [35] and Tanzania [36], two countries bordering the DRC, it would be prudent that a continuous and regular monitoring of the occurrence of this haplotype be conducted.

The SP was quickly removed for malaria first-line-treatment in malaria endemic areas when many reports showed strong resistance rates. Studies conducted in the DRC have also confirmed it, but with important inter-sites and inter-studies variations [15–18]. It is not easy to say that SP resistance is declining or increasing because there were not two repetitive studies conducted in the same place, with the same methodology and at different time. Besides, it appears that SP resistance is more pronounced in the East than the West of the DRC. SP is nevertheless still used for intermittent preventive treatment in pregnant women (IPTp) and none of these studies assessed the impact of the use of this molecule in high resistance area.

At the time of the use of ACT, it is very important to monitor the emergence of artemisinin resistant strains in Africa. Indeed, this is the first time that a resistance molecular marker is identified before the resistant strains spreads to all malaria endemic regions. Actually, two studies were related to DRC samples on the *k13*-*propeller* gene [21, 24]. None of polymorphisms identified in Southeast Asia have been found in the DRC but new mutations on *k13* have been discovered in Africa. Further studies should be conducted to assess the impact of these new mutations on susceptibility to artemisinin.

## Conclusion

Only 13 studies were identified in this review and the majority of them were not representative of the whole country. So their results are not generalizable. DRC is a very large country and malaria responses to drugs are very different from one place to another. Ministry of Health must implement a national surveillance system for monitoring malaria drug resistance and this surveillance should be conducted frequently, in many sentinel sites and this especially to react as fast as the occurrence of artemisinin-resistant or AMQ-resistant strains in a part of the DRC.
